# Characterization of *Anopheles* Species and Entomological Indicators Following Indoor Residual Spraying Campaign in Cuando Cubango, Angola

**DOI:** 10.3390/insects16090892

**Published:** 2025-08-26

**Authors:** André Domingos, Ana Direito, Gonçalo Alves, Paulo Máquina, Cani P. Jorge, José F. Martins, Lizette L. Koekemoer, Sergio Lopes, Luzala Garcia

**Affiliations:** 1 National Malaria Control Programme Ministry of Health, Luanda, Angola; andrejosedomingos@gmail.com (A.D.); canipedro@yahoo.fr (C.P.J.); jose.martins8219@gmail.com (J.F.M.); 2 The MENTOR Initiative, Haywards Heath RH16 1PG, UK; anasofiadireito@gmail.com; 3 Global Health and Tropical Medicine GHTM, Associate Laboratory in Translation and Innovation Towards Global Health LA-REAL, Instituto de Higiene e Medicina Tropical, Universidade Nova de Lisboa UNL, 1349-008 Lisboa, Portugal; 4 SADC Malaria Elimination Eight Secretariat, Windhoek, Namibia; pquim1981@gmail.com; 5 Wits Research Institute for Malaria, Faculty of Health Sciences, University of the Witwatersrand, Johannesburg 2000, South Africa; lizette.koekemoer@wits.ac.za; 6 Centre for Emerging Zoonotic & Parasitic Diseases, National Institute for Communicable Diseases, Division of the National Health Laboratory Service, Johannesburg 2192, South Africa

**Keywords:** *Anopheles funestus*, *Anopheles gambiae*, *Anopheles arabiensis*, *Plasmodium falciparum*, indoor residual spraying, malaria, Cuando Cubango, Angola

## Abstract

Malaria remains a significant public health challenge in Angola, particularly in Cuando Cubango province. This study aimed to characterize the local *Anopheles* mosquito population, evaluate the impact of indoor residual spraying (IRS) on key entomological indicators, and assess the community knowledge, attitudes, and practices (KAP) related to malaria and vector control. Mosquito collections were conducted indoors at three sites over five months using CDC light traps and Prokopack aspirators. Ten *Anopheles* species were identified, with *An. funestus* s.s. being the predominant vector, accounting for 91.7% of the *Funestus* group. The overall *Plasmodium falciparum* circumsporozoite protein (CSP) infection rate was 9.2%, with Makua exhibiting the highest rate (10.2%). Following IRS, the indoor resting density of the *Funestus* group decreased significantly in Makua and Agostinho Neto. In Makua, *An. funestus* s.s. CSP infection rates decreased by 55% following IRS implementation; however, this reduction was not statistically significant. Knockdown resistance mutations were detected in *An. arabiensis* and *An. gambiae* s.s. The 2020/2021 IRS campaign achieved 95% spray coverage and reached 421,856 individuals. Complementary KAP surveys with 647 households showed that 87% of respondents correctly identified mosquitoes as malaria vectors, 78% recognized key symptoms, and 89% supported continued IRS. Reported adherence to post-IRS precautions exceeded 70% and preference for IRS over ITN was expressed by most households. These findings highlight both biological and social dimensions of malaria control, demonstrating that vector control, community acceptance, and entomological surveillance are essential for effective and sustainable malaria interventions in Cuando Cubango.

## Simple Summary

Malaria is a deadly disease spread by female *Anopheles* mosquitoes that affects millions of people. Angola has one of the highest malaria burdens in the world, making it a serious public health problem. This study aimed to understand which mosquito species carry malaria parasites, whether spraying the inside walls of houses with insecticides impacts entomological indicators, and how communities perceive and use these prevention measures in Cuando Cubango province. Researchers collected mosquitoes from three locations over five months and interviewed local households after the spraying campaign. The results showed that *An. funestus* s.s. was the primary malaria vector. After spraying houses with insecticides, there were fewer mosquitoes and lower rates of malaria infection in mosquitoes, particularly in one location where infection rates dropped by 60 percent. However, this reduction was not consistent across all areas studied. Most community members demonstrated good knowledge about malaria, supported IRS, and followed advice on how to protect their homes after spraying. These findings provide important information for malaria control programs in Angola, help health authorities to understand which mosquitoes to target, and demonstrate that house spraying can be effective in reducing malaria transmission, although additional control measures are needed for maximum impact.

## 1. Introduction

Malaria remains a significant global public health threat, with approximately 263 million cases and 597,000 fatalities reported worldwide in 2023. Angola bears a substantial portion of this burden, accounting for 3.1% of global malaria cases and 2.7% of related deaths [1]. In 2023, Angola recorded a malaria incidence rate of 260 per 1000 population at risk. This figure represents an increase of between 25% and 63% compared to the 2015 baseline. Angola is among the countries in the WHO African region where the estimated case incidence was higher in 2023 than in 2015 [1]. Despite these figures, Angola made significant strides in reducing its malaria mortality rate from 58.2% in 2016 to 26.3% in 2019 [2].

Most malaria cases in Angola are caused by *Plasmodium falciparum*, which remains the dominant species across the country [3]. However, molecular studies carried out in the last two decades have demonstrated the presence of *P. malariae*, *P. ovale*, and *P. vivax* in smaller proportions [4,5].

In Angola, malaria transmission is complex, with the presence of multiple vectors, including *Anopheles gambiae* sensu stricto (s.s.), *An. coluzzii*, *An. arabiensis*, and *An. funestus* s.s. [2,6,7,8,9]. Members of the *Funestus* group, previously confirmed as secondary malaria vectors in other regions, have been recently reported in Angola [9], highlighting the need for further investigation into the lesser-studied species of this group. Vector control is primarily made through the distribution of insecticide-treated mosquito nets (ITN) and indoor residual spraying (IRS). However, the effectiveness of these interventions can be compromised by the emergence of knockdown resistance (kdr), which is associated with pyrethroids and DDT insecticides. Kdr mutations, particularly West African L1014F and East African L1014S, have already been documented in *An. gambiae* complex populations in various locations in Angola [9,10], causing a significant threat to the efficacy of ITN and IRS [11,12,13].

Cuando Cubango province was previously classified as mesoendemic with unstable malaria transmission. According to the updated malaria risk stratification map, four districts in the province are now classified as having moderate transmission, two as having high transmission, and three as having very high transmission [2]. In 2018, the prevalence of malaria in the province was 38%, the second highest in the country [14]. To address the challenges of the province, the National Malaria Control Program (NMCP), in partnership with international partners, intensified efforts to control and eliminate malaria in southern Angola. Since 2018, a large-scale IRS campaign has been implemented in the province with support from the Elimination 8 Initiative (E8). The E8 focused on reducing malaria transmission within Angola and strengthening elimination efforts in the neighboring Republic of Namibia through coordinated cross-border interventions, enhanced surveillance, and expanded access to malaria services in border areas [15]. NMCP, in collaboration with the Mentor Initiative, has implemented IRS activities with Actellic^®^ 300 CS across border districts in southern Angola, including the four border districts of Cuando Cubango. During the 2019/2020 campaign, 13,047 structures were sprayed across the districts of Calai, Cuangar, Dirico, and Rivungo, protecting 57,157 people and achieving a coverage of 97.8%. In addition to IRS, an ITN campaign was conducted in the province in 2018, during which 286,543 ITNs were distributed, covering 98.5% of the population. From 2018, operational research studies and field assessments have been conducted to evaluate the coverage, effectiveness, and overall impact of these interventions [14]. Nevertheless, limited information is available on the composition of malaria vector species, *P. falciparum* infection rates, host preferences, and the presence of kdr mutations in Cuando Cubango province. A detailed understanding of these entomological parameters is essential for designing effective, evidence-based, and geographically tailored vector control strategies. This study aimed to update the entomological profile of malaria vectors in the province and to assess the impact of the 2020/2021 IRS campaign on entomological indicators.

## 2. Materials and Methods

### 2.1. Study Area

The study was conducted in Cuando Cubango province, located in southeastern Angola. Cuando Cubango shares borders with the Republic of Namibia to the south, with the Republic of Zambia to the east and with the Angolan provinces of Moxico to the north, Bié to the northwest, Huíla to the west, and Cunene to the southwest. The province covers an area of approximately 199,049 km^2^ and had a population of 534,002 [16]. The average elevation of the province is around 1200 m above sea level. Major rivers crossing the province include the Cuando, Cubango, and Cuito rivers [17]. The average temperature in Cuando Cubango varies between 16.3 °C and 25.5 °C, with a rainy season from October to April and a dry season from May to September.

### 2.2. Cuando Cubango IRS Campaign

**Figure 1:**
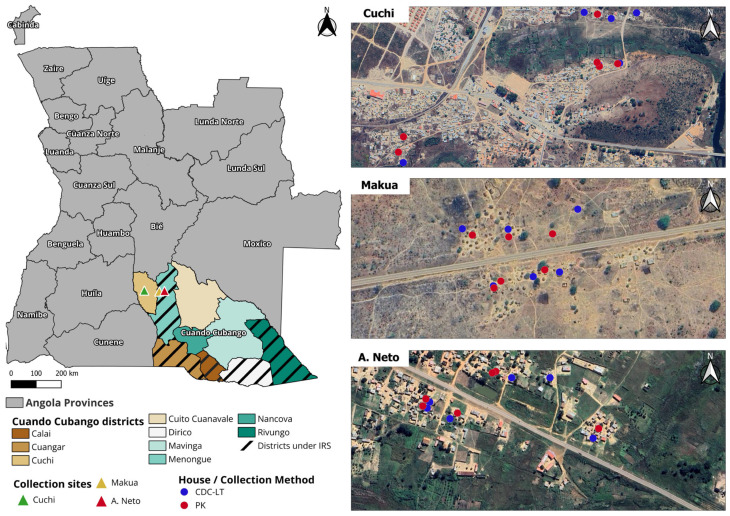
Mosquito collection sites and district under IRS.

The Cuando Cubango IRS campaign ran from December 2020 to February 2021 in the four border districts of Calai, Cuangar, Dirico, and Rivungo, according to the NMCP’s strategic plan. In this campaign, the Menongue district was also included (Figure 1). A total of 1519 individuals were trained, including spray operators (551), enumerators (551), mobilizers (250), data collectors (65), and team leaders and supervisors (102). Training followed the regional Southern Africa Development Community Malaria Elimination Eight Secretariat (SADAC-MEES) guidelines and included modules on insecticide handling, safety, spray technique, data collection, and community sensitization [18]. The selection of sprayable structures was conducted in accordance with previous guidelines. Actellic^®^ 300 CS (Syngenta Crop Protection AG, Basel, Switzerland; active ingredient: pirimiphos-methyl) was applied at a dosage of 1 g/m^2^. Structure enumeration, microplanning, and real-time monitoring were carried out using the pilot version of Reveal v.1 (Akros Inc., Lusaka, Zambia). Data were collected using mobile devices, synchronized through the Reveal platform, and used to generate automated reports on campaign progress and coverage.

### 2.3. Post-IRS Knowledge, Attitudes, and Practices Survey

A community-based cross-sectional Knowledge, Attitudes, and Practices (KAP) study was conducted between February and March 2021 in the districts of Calai, Cuangar, Dirico, and Menongue, following completion of the IRS campaign. The survey was administered in Portuguese to ensure accessibility and accurate responses from participants. Households were selected using a two-stage randomized cluster sampling method. One adult respondent per household was interviewed using a structured questionnaire that had been previously piloted in Menongue. The questionnaire assessed knowledge of malaria transmission and symptoms; attitudes towards IRS and malaria prevention; and household practices, including ITN use, IRS compliance, and environmental risk factors. Direct observations were made regarding housing conditions and IRS use. Data were collected using KoboToolbox https://www.kobotoolbox.org (accessed on 24 August 2025), validated daily, and analyzed using Microsoft Excel 365 (Version 2507) (Build 16.0.19029.20136). Ethical approval was obtained from the Angolan National Ethics Committee.

### 2.4. Entomological Surveillance and Mosquito Collections

Two districts were selected for the entomological survey: Menongue (targeted for IRS) and Cuchi (no IRS intervention) (Figure 1). Collection sites were chosen based on logistical capacity and IRS campaign plans. Adult mosquitoes were collected from three sites: two in the district of Menongue, Makua (−14.679500, 17.453611) and Agostinho Neto (−14.655028, 17.646500; A. Neto); and one in the district of Cuchi, Cuchi (−14.649667, 16.898083). At each site, twelve houses were randomly selected for adult mosquito collections and equally assigned to one of the sampling methods. Informed consent was obtained from the head of each household, and collection procedures were clearly communicated to all household members. Mosquitoes were collected monthly over three consecutive nights from November 2020 to March 2021. Indoor host-seeking mosquitoes were captured using CDC light traps (CDC-LT; Model 512; John W. Hock Company, Gainesville, FL, USA). Traps were placed at the foot end of occupied sleeping areas, approximately 1.5 m above the ground, and operated from 18:00 to 07:00. Indoor resting mosquitoes were captured using Prokopack aspirators (PK; Model 1419, John W. Hock Company, Gainesville, FL, USA). Indoor aspirations were conducted in the morning from 07:00 to 09:00 by two collectors. Each house was examined for 15 min, with special attention paid to areas where people had slept the previous night. PK collection cups were replaced every 5 min to prevent damage to the aspirated mosquitoes. Collected mosquitoes were transported to the field insectary for morphological identification. Larval collections were conducted across multiple breeding sites across both districts using the standard dipping method. Larvae were transferred to the field insectary and reared to adulthood. At 48 h post-emergence, the adults were killed in an alcohol chamber and subjected to morphological identification.

### 2.5. Morphological Identification

Female *Anopheles* mosquitoes were identified using well-established morphological keys [19,20]. Morphological identifications were carried out by trained entomologists in the field insectary. Abdomens from female *Anopheles* mosquitoes were categorized as fed, unfed, gravid, and half-gravid. Samples were stored individually in labeled microtubes containing blue indicator silica gel at room temperature.

### 2.6. Molecular Species Identification of Anopheles Mosquitoes

*An. gambiae* complex and *Funestus* group members were identified to species level using molecular methods. Genomic DNA was individually extracted from legs [21]. Molecular identification of *An. gambiae* complex members was performed using known protocols [22,23]. Members of the *Funestus* group were identified through a multiplex PCR assay to separate *An. funestus* s.s. from the more zoophilic species *An. parensis*, *An. rivulorum*, *An. rivulorum-like*, *An. leesoni*, and *An. vaneedeni* [21,24]. Negative controls included a PCR master mix without a DNA template and a DNA extraction negative control. Positive controls comprised samples with previously established species molecular identification.

### 2.7. Detection of Plasmodium falciparum Circumsporozoite Protein

Head and thoraces of unfed female *Anopheles* mosquitoes were analyzed for the presence of *P. falciparum* circumsporozoite protein (CSP) using enzyme-linked-immunosorbent assay (ELISA) [25]. If a mosquito tested positive, a confirmatory ELISA test was performed using a boiling step [26]. Mosquitoes were considered positive for *P. falciparum* CSP if both tests yielded positive results.

### 2.8. Blood Meal Origin

Abdomens of blood-fed *Anopheles* mosquitoes were subjected to multiplex-PCR targeting the cytochrome B gene for the source of the blood meal [22]. Five potential hosts were tested: human, cow, goat, pig, and dog.

### 2.9. Detection of Knockdown Resistance

Knockdown resistance mutations were investigated in *An. arabiensis* and *An. gambiae* s.s. following protocols targeting kdr-West (L1014F) and kdr-East (L1014S) mutations [27,28,29].

### 2.10. Entomological Parameters and Statitical Analysis

Entomological parameters were assessed before (November 2020 to January 2021) and after (February to March 2021) IRS. The *P. falciparum* infection rate (IR%) was calculated as the percentage of unfed mosquitoes testing positive for CSP in the head-thorax (IR% = No. of CSP-positive head-thorax samples/total No. of head-thoraces analyzed × 100). The human blood index (HBI) was calculated as the proportion of blood-fed anopheline mosquitoes that fed on humans, including those with mixed blood meals (HBI = No. of human-fed mosquitoes/total No. of blood-fed mosquitoes analyzed × 100). The adjusted HBI was used to compensate for unidentified blood meal sources, excluding mosquitoes with unidentified blood meals from the formula [30]. Vector density (VD) was calculated as the number of *Funestus* group members per night per CDC-LT per month. Indoor resting density (IRD) was calculated as the number of *Funestus* group members collected resting indoors per house (IRD = No. of *Funestus* group members collected indoor by PK/No. of surveyed houses). To assess the impact of IRS on entomological parameters, we conducted a before-and-after analysis at the A. Neto and Makua collection sites, comparing IRD, VD, and IR% pre- and post-IRS. The Chi-square test was used to compare proportions, with a significance level set at 0.05.

### 2.11. Ethics Approval and Consent to Participate

The entomological and KAP studies received approval from the Instituto Nacional de Investigação em Saúde de Angola (INIS), under approval letters 05/2021 and 06/2021, respectively.

## 3. Results

### 3.1. Cuando Cubango IRS Campaign and Knowledge, Attitudes, and Practices Survey

The 2020/2021 IRS campaign was conducted from December 2020 to February 2021 across the districts of Rivungo, Dirico, Calai, Cuangar, and Menongue, covering 17 communes (Table 1). A total of 118,828 structures were identified, of which 113,052 were successfully sprayed, achieving a coverage rate of 95.14%. The campaign reached 421,856 individuals, an increase of 638% compared to the previous campaign, primarily due to the inclusion of Menongue district.

Regarding the KAP survey, a total of 647 heads of households were interviewed. Among respondents, 92.4% reported having received IRS in the previous year. Additionally, 87% correctly identified mosquitoes as the primary vectors of malaria, and 78% recognized key symptoms such as fever and chills. Attitudes toward IRS were positive, with 89% of participants supporting its continued implementation. Reported adherence to post-IRS precautions, such as refraining from washing treated walls, exceeded 70%. Only 12% of respondents expressed a preference for ITNs over IRS, primarily due to the perceived longer-lasting protection offered by IRS. Direct observations indicated that, in fewer than 80% of sprayed households, IRS marks remained visible and insecticide-treated surfaces were intact, suggesting moderate retention of treatment indicators.

### 3.2. Anopheles Species Composition

**Figure 2:**
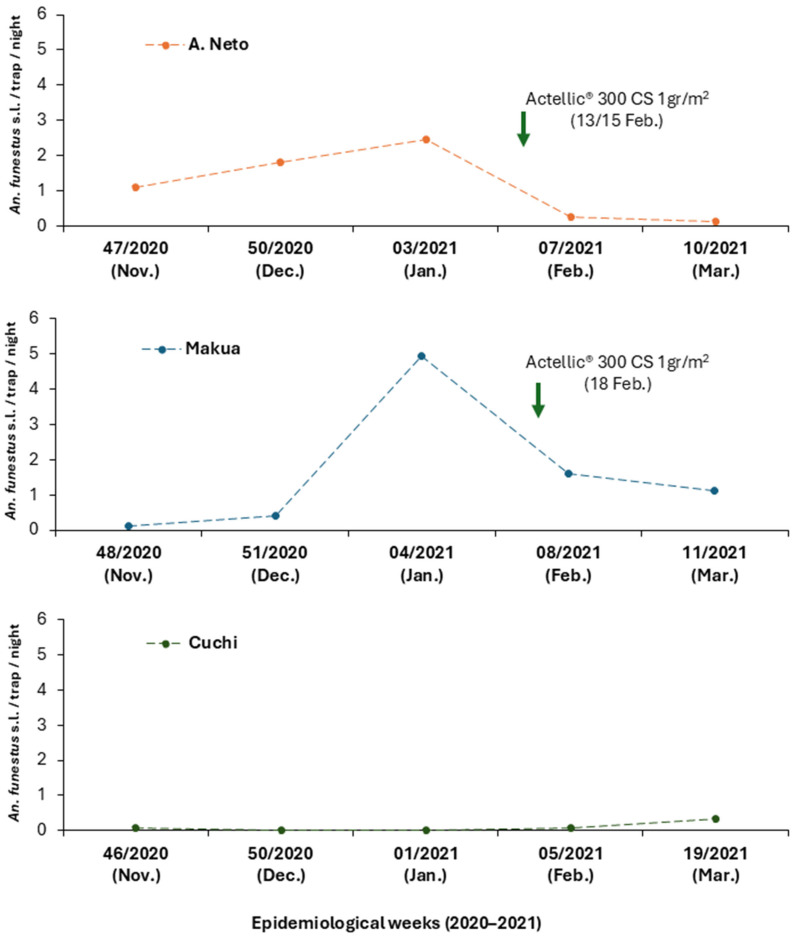
Effect of Actellic^®^ 300 CS on *An. funestus* s.l. VD across the three collection sites (A. Neto, Makua, and Cuchi) over epidemiological weeks from November 2020 to March 2021.

A total of 1436 adult mosquitoes were collected across the three sampling sites. Of these, 549 (38.2%) were female and 20 (1.4%) were male *Anopheles*. Among the females, 86.1% were captured using CDC-LT (Table 2). Unfed *Anopheles* comprised the majority of the collections (74.9%), followed by blood-fed females (23.9%) (Table 2). Makua accounted for 51.5% of all adult *Anopheles* mosquitoes (Table 3). In total, ten *Anopheles* species were identified. The *Funestus* group was the most prevalent, accounting for 91.4% of all *Anopheles* collected, followed by *An. gambiae* complex, *An. rufipes*, *An. squamosus*, *An. concolor*, and *An. ruarinus* (Table 3). Species-specific identification of a subsample of the *Funestus* group revealed the presence of multiple members. The majority were identified as *An. funestus* s.s. (91.7%), followed by *An. vaneedeni*, *An. leesoni*, *An. rivulorum*, and *An. rivulorum-like* (Table 4). The remaining 26 specimens failed to amplify. Regarding the *An. gambiae* complex, ten were identified as *An. arabiensis*, while four failed to amplify (Table 4). In A. Neto and Makua, a rising VD was observed from November 2020 to January 2021, followed by a decrease after IRS (Figure 2). In Cuchi, the VD was constantly low, increasing from January to March (Figure 2). When comparing the periods before and after IRS (Table 5), we observed in A. Neto a significant decrease in VD from 2.03 mosquitoes/trap/night pre-IRS to 0.18 mosquitoes/trap/night post-IRS (χ^2^, *p* < 0.0001). In Makua, no significant difference was observed, with VD increasing slightly from 0.99 to 1.36 mosquitoes/trap/night (χ^2^, *p* > 0.05). In Cuchi, a significant increase in VD was recorded, rising from 0.02 to 0.22 mosquitoes/trap/night (χ^2^, *p* < 0.01). For the *Funestus* group IRD, the results showed a significant decrease at both Makua and A. Neto after IRS intervention (χ^2^ test, *p* < 0.001) (Table 6). By contrast, a non-significant increase in the *Funestus* group IRD was observed in Cuchi (Fisher, *p* > 0.05; Table 6).

### 3.3. Origin of Blood Meals

A total of 106 blood-engorged *Anopheles* mosquitoes were analyzed to determine the origin of their blood meals (Table 7). Among these, 18.9% had fed on humans, indicating a preference for human hosts overall. *An. funestus* s.s. exhibited the highest adjusted HBI, with 78.6% in A. Neto and 57.1% in Makua, resulting in an overall adjusted HBI of 67.9%. By contrast, *An. arabiensis* showed a lower adjusted HBI of 33.3%, feeding more frequently on cattle rather than on humans. *An. rufipes* demonstrated no clear host preference, with an adjusted HBI of 50%.

### 3.4. Plasmodium falciparum Infection Rate

Head-thoraces from 250 unfed female *Anopheles* mosquitoes were analyzed using ELISA for the presence of CSP (Table 8). Among these, 23 tested positive, corresponding to an overall infection rate of 9.2% (23/250). CSP-positive mosquitoes were detected exclusively as *An. funestus* s.s., which exhibited a species-specific infection rate of 9.5%. By collection site, Makua had the highest CSP infection rate at 10.2%, followed by A. Neto with 9.3%. No CSP-positive mosquitoes were detected in Cuchi. When considering only *An. funestus* s.s., the infection rates were 10.5% in Makua and 9.4% in A. Neto. Before IRS, 18 *An. funestus* s.s. were CSP positive, yielding a pre-IRS IR of 10.2%. Infection rates were higher in Makua (14.0%) compared to A. Neto (8.3%), although not statistically significant (Fisher’s exact test, *p* > 0.05). Following IRS intervention, the overall IR decreased to 8.9%, representing a 12.7% reduction; however, it was not statistically significant (*p* > 0.05). In Makua, the IR dropped to 6.3%, a 55.0% reduction from pre-IRS levels, though not statistically significant (*p* > 0.05). Conversely, in A. Neto, the IR increased to 25.0%, representing a 201% rise compared to the pre-IRS period (Table 9).

### 3.5. Detection of Knockdown Resistance

*Anopheles gambiae* complex mosquitoes were genotyped for the presence of L1014F and L1014S kdr (Table 10). In Menongue, all *An. arabiensis* specimens (*n* = 3) were homozygous susceptible (SS) for both L1014F and L1014S alleles. In Cuchi, all 33 *An. arabiensis* were SS for L1014F, and five were heterozygous (RS) for L1014S. For *An. arabiensis*, no homozygous resistant genotypes (RR) were detected in *An. arabiensis* for either allele. The overall resistant allele frequency for L1014F in *An. arabiensis* was 0.0, while the frequency for L1014S was 0.069. By contrast, all five *An. gambiae* s.s. from Menongue were homozygous resistant (RR) for the L1014F allele, resulting in a resistant allele frequency of 1.0. No resistance alleles were detected for L1014S in *An. gambiae* s.s.

## 4. Discussion

This study provides valuable insights into the *Anopheles* mosquito population and the impact of IRS on entomological indicators in Cuando Cubango. Our results confirm the presence of primary malaria vectors such as *An. funestus* s.s., *An. gambiae* s.s., and *An. arabiensis*, consistent with previous reports from Angola and other regions of sub-Saharan Africa [6,8,9,20]. In addition, we confirm the presence of *An. concolor*, *An. ruarinus*, *An. rufipes*, and *An. squamosus* [8,19] and report for the first time, through molecular methods, the presence of *An. leesoni*, *An. rivulorum*, *An. rivulorum-like*, and *An. vaneedeni* within the *Funestus* group. With the exception of *An. concolor* and *An. ruarinus*, all other species reported were found to be naturally infected with *P. falciparum* in other sub-Saharan African countries [31,32,33,34]. These findings underscore the urgent need to accelerate research on the role of lesser-studied mosquito species in malaria transmission within the country.

*An. funestus* s.s. was identified as the primary malaria vector in the study area, consistent with findings from Angola, Zambia, and Namibia [9,35,36]. The absence of *An. gambiae* s.s. in indoor collections, despite its detection at the larval stage, may reflect behavioral adaptations to vector control interventions such as ITNs and IRS. Shifts toward exophilic and exophagic behaviors have been reported elsewhere [37].

Interestingly, *An. arabiensis* showed a preference for feeding on cattle, reflecting its opportunistic behavior and tendency to feed on both humans and animals [38]. This observation is particularly relevant, as the presence of cattle may contribute to zooprophylaxis, diverting mosquito bites away from humans [39]. However, due to the small sample size in this study, further research is needed to better understand the feeding preferences and host-seeking behavior of *An. arabiensis* in this setting. Additionally, we observed a high number of cases with no amplification of target DNA, for which no specific cause was identified. Possible explanations include degraded DNA or blood meals from non-human hosts not covered in our analysis. These findings underscore the importance of expanding host identification efforts and improving sample quality to strengthen future entomological assessments.

Our study confirms the fixation of the West African kdr mutation L1014F in *An. gambiae* s.s. from Menongue and reports the presence of the East African kdr mutation L1014S in *An. arabiensis* from the Cuchi district. To our knowledge, this is the first time that both West and East African kdr mutations have been investigated in this province. These findings reveal an unreported challenge in the management of insecticides for public health use and underscore the critical need for regular monitoring of insecticide resistance patterns in local vector populations.

The geographic scope of this study was limited; therefore, expanding entomological surveillance to other districts within Cuando Cubango is essential to capture spatial heterogeneity in vector populations and to better inform decision makers.

The IRS campaign achieved a coverage rate of 95%, protecting 421,856 individuals, a level of coverage that is critical given that IRS effectiveness is strongly associated with achieving at least 80% household coverage [40]. However, some studies have shown that lower coverage levels can still produce protective effects comparable to campaigns reaching or exceeding the 80% threshold [41,42], Conversely, other evidence indicates that coverage below 80% may fail to generate the desired protective outcomes. This variability highlights the influence of local factors, such as transmission intensity, vector behavior, and housing conditions, on the effectiveness of IRS interventions [42].

Community acceptance of IRS was high, with 89% of respondents supporting continued spraying and over 70% adhering to post-spray precautions. This level of engagement likely contributed to the observed reductions in vector density and infection rates, particularly in Makua. The preference for IRS over ITNs, cited by 88% of respondents, highlights the perceived value of IRS in providing long-lasting protection.

Post-IRS entomological indicators showed a significant reduction in the indoor resting density (IRD) of the *Funestus* group in both Makua and Agostinho Neto. Vector density also declined significantly in Agostinho Neto but not in Makua. The lack of statistically significant reductions in CSP infection rates may be attributed to several post-IRS factors, such as the short observation period (only two collection rounds), small sample sizes of mosquitoes collected and analyzed, and environmental variability, such as below-average rainfall, which may have independently suppressed mosquito populations. These limitations underscore the importance of extended surveillance periods and larger sample sizes to robustly evaluate intervention impact.

## 5. Conclusions

Our study provides key insights into malaria vector populations and the impact of IRS in Cuando Cubango, demonstrating its effectiveness in reducing *Anopheles* density and its potential to lower transmission. However, small sample sizes, limited post-intervention data, and the absence of epidemiological indicators constrain the strength of conclusions regarding IRS impact. To address these gaps, we recommend integrated studies that combine entomological and epidemiological data for a more robust evaluation of vector control interventions. Sustained surveillance, community engagement, and accurate species identification [43] are essential to guide locally tailored and adaptive strategies. Further research is needed to clarify the role of *An. gambiae* s.s. and members of the *Funestus* group in malaria transmission. These findings underscore the importance of comprehensive knowledge to support evidence-based, locally tailored, and sustainable malaria vector control strategies.

## Figures and Tables

**Table 1:** Cuando Cubango 2020/2021 IRS campaign results.

District	Commune	Sprayed Structures	Coverage (%)	No. People Protected
Rivungo	Rivungo sede	2959	91	12,056
	Luiana	2474	85	11,201
	Tchipundo	3352	99	14,777
	Neriquinha	247	82	952
	Subtotal	9032	92	38,986
Dirico	Dirico sede	1459	94	4725
	Mucusso	663	99	2683
	Xamavera	976	88	3973
	Subtotal	3098	93	11,381
Calai	Calai Sede	3280	92	11,576
	Mavengue	284	97	866
	Mawé	282	98	1066
	Subtotal	3846	92	13,508
Cuangar	Cuangar sede	3302	94	11,806
	Bondo-Caila	1608	88	6159
	Savate	1801	91	5483
	Subtotal	6711	95	23,448
Menongue	Menongue sede	81,584	96	301,143
	Missombo	1809	98	6651
	Caiundo	5390	91	22,338
	Jamba Cueio	1582	98	4401
	Subtotal	90,365	96	334,533
Total		113,052	95	421,856

**Table 2:** Distribution of *Anopheles* females by abdominal status and collection method.

Collection Method	Unfed (%)	Fed (%)	Half Gravid (%)	Gravid (%)	Unknown (%)	Total (%)
CDC-LT	396 (96.4)	76 (58.0)	2 (100.0)	1 (25.0)	1 (100.0)	476 (86.1)
PK	15 (3.6)	55 (42.0)	0 (0.0)	3 (75.0)	0 (0.0)	73 (13.3)
Total	411 (74.9)	131 (23.9)	2 (0.4)	4 (0.7)	1 (0.2)	549

**Table 3:** *Anopheles* mosquito composition, by collection site and method.

Collection Site	Collection Method	*An. concolor*	*Funestus* Group	*An. gambiae* Complex	*An. ruarinus*	*An. rufipes*	*An. squamosus*	*An*. spp. *	Total
A. Neto	CDC-LT	0	210	0	0	0	0	2	212
	PK	0	33	0	0	0	0	0	33
	Subtotal	0	243	0	0	0	0	2	245
Makua	CDC-LT	2	215	5	1	9	11	1	244
	PK	0	35	1	0	3	0	0	39
	Subtotal	2	250	6	1	12	11	1	283
Cuchi	CDC-LT	0	10	8	0	1	0	1	20
	PK	0	1	0	0	0	0	0	1
	Subtotal	0	11	8	0	1	0	1	21
Total (%)		2 (0.4)	504 (91.8)	14 (2.6)	1 (0.2)	13 (2.4)	11 (2.0)	4 (0.7)	549

* *Anopheles* mosquitoes not morphologically identified due to lack or damage of morphological features.

**Table 4:** Molecular identification of adults of *An. gambiae* s.l. and *An. funestus* s.l.

Mosquito Species	*N*	Species-Specific PCR Identification	Sentinel Site			Total (%)
			A. Neto	Makua	Cuchi	
*An. gambiae* complex	14	*An. arabiensis*	0	2	8	10 (71.4)
		Failed identification	0	4	0	4 (28.6)
*Funestus* group	375	*An. funestus* s.s.	176	159	9	344 (91.7)
		*An. leesoni*	0	1	0	1 (0.3)
		*An. rivulorum*	1	0	0	1 (0.3)
		*An. rivulorum-like*	0	1	0	1 (0.3)
		*An. vaneedeni*	0	2	0	2 (0.5)
		Failed identification	8	16	2	26 (6.9)

**Table 5:** Impact of IRS on *Funestus* group VD per collection site.

Collection Sites	Pre-IRS (Nov-20 to Jan-21)			Post-IRS (Feb-21 to Mar-21)			χ^2^, *p*-Value
	No. Captures	Trapping Effort	VD	No. Captures	Trapping Effort	VD	
Cuchi	1	61	0.02	17	78	0.22	<0.01
Makua	113	114	0.99	102	75	1.36	>0.05
A. Neto	195	96	2.03	16	87	0.18	<0.0001

**Table 6:** Impact of IRS on *Funestus* group IRD per collection site.

Collection Sites	Pre-IRS (Nov-20 to Jan-21)			Post-IRS (Feb-21 to Mar-21)			χ^2^, *p*-Value
	No. Captures	Trapping Effort	IRD	No. Captures	Trapping Effort	IRD	
Cuchi	0	29	0.00	1	9	0.11	>0.05 ^a^
Makua	34	49	0.69	1	15	0.07	<0.001
A. Neto	33	40	0.83	0	20	0.0	<0.001

^a^ Fisher Exact test.

**Table 7:** Host preferences of *Anopheles* mosquitoes in Cuchi, Agostinho Neto, and Makua.

Mosquito Species	Collection Site	*N*	Blood Meal Source				HBI	Adjusted HBI
			Cow	Goat	Human	N/A		
*An. funestus* s.s.	Agostinho Neto	48	1	2	11	34	22.9	78.6
	Makua	54	6	0	8	40	14.8	57.1
	Subtotal	102	7	2	19	74	18.6	67.9
*An. arabiensis*	Cuchi	3	1	0	1	1	33.3	50.0
	Makua	1	1	0	0	0	0.0	0.0
	Subtotal	4	2	0	1	1	25.0	33.3
*An. rufipes*	Cuchi	1	1	0	0	0	0.0	0.0
	Makua	3	1	0	2	0	66.7	66.7
	Subtotal	4	2	0	2	0	50.0	50.0
Total (%)		106	9 (8.5)	2 (1.9)	20 (18.9)	75 (70.8)	-	-

N/A: no amplification of target DNA.

**Table 8:** Unfed *Anopheles* mosquito head-thoraces tested for CSP.

Mosquitoes	Cuchi			A. Neto			Makua			Total		
	Tested	CSP+	IR	Tested	CSP+	IR	Tested	CSP+	IR	Tested	CSP+	IR%
*An. arabiensis*	5	0	0.0	0	0	0.0	1	0	0.0	6	0	0.0
*An. funestus* s.s.	8	0	0.0	128	12	9.4	105	11	10.5	241	23	9.5
*An. rivulorum*	0	-	-	1	0	0.0	0	-	-	1	0	0.0
*An. leesoni*	0	-	-	0	-	-	1	0	0.0	1	0	0.0
*An. vaneedeni*	0	-	-	0	-	-	1	0	0.0	1	0	0.0
Total	13	0	0.0	129	12	9.3	108	11	10.2	250	23	9.2

CSP+: number of head-thoraces positive for *P. falciparum* circumsporozoite protein.

**Table 9:** IR% of *An. funestus* s.s. from A. Neto and Makua.

Month-Year	Collection Sites						Total		
	A. Neto			Makua					
	Tested	Positive	IR%	Tested	Positive	IR%	Tested	Positive	IR%
Nov-20	9	0	0.0	4	0	0	13	0	0.0
Dec-20	45	4	8.9	9	0	0	54	4	7.4
Jan-21	66	6	9.1	44	8	18.2	110	14	12.7
Total pre-IRS	120	10	8.3	57	8	14.0	177	18	10.2
Feb-21	8	2	25.0	27	2	7.4	35	4	11.4
Mar-21	0	-	-	21	1	4.8	21	1	4.8
Total post-IRS	8	2	25.0	18	3	6.3	56	5	8.9

**Table 10:** Genotype frequencies of L1014F and L1014S for *An. arabiensis* and *An. gambiae* s.s.

Mosquitoes	District of Collection	*N*	Genotype L1014F			Genotype L1014S			Resistant Allele Frequence	
			SS	RS	RR	SS	RS	RR	F (Phe)	F (Ser)
*An. arabiensis*	Menongue	3 (1)	3	0	0	3 (1)	0	0	0.0	0.0
	Cuchi	33 (25)	33 (25)	0	0	28 (20)	5 (5)	0	0.0	0.076
	Subtotal	36 (26)	36 (25)	0	0	31 (21)	5 (5)	0	0.0	0.069
*An. gambiae* s.s.	Menongue	5 (5)	0	0	5 (5)	5 (5)	0	0	1.0	0.0

Numbers in parentheses indicate larvae tested for kdr genotypes out of the total sample size (N); SS = homozygote susceptible, RS = heterozygote resistance, RR = homozygote resistance; S = susceptible wildtype allele, R = resistance allele (L1014F/L1014S mutations).

## Data Availability

The data and materials that support the findings of this study are available upon request from the corresponding authors.

## Abbreviations

The following abbreviations are used in this manuscript:

IRS	Indoor Residual Spraying
ITN	Insecticide-Treated Nets
KAP	Knowledge, Attitudes, and Practices
kdr	Knockdown Resistance
MI	The Mentor Initiative
NMCP	National Malaria Control Program
SA-DAC-MEES	Southern Africa Development Community Malaria Elimination Eight Secretariat
